# Genomic *in situ* hybridization identifies parental chromosomes in hybrid scallop (Bivalvia, Pectinoida, Pectinidae) between female *Chlamys farreri* and male *Argopecten irradians irradians*

**DOI:** 10.3897/CompCytogen.v9i2.8943

**Published:** 2015-05-20

**Authors:** Xiaoting Huang, Ke Bi, Wei Lu, Shi Wang, Lingling Zhang, Zhenmin Bao

**Affiliations:** 1 Key Laboratory of Marine Genetics and Breeding (Ocean University of China), Ministry of Education, Qingdao 266003, China; 2 Museum of Vertebrate Zoology, University of California, Berkeley, California 94720, USA

**Keywords:** Scallop, interspecific hybridization, GISH, karyotype, chromosome aberration

## Abstract

Interspecific crossing was artificially carried out between *Chlamys farreri* (Jones & Preston, 1904) ♀ and *Argopecten irradians irradians* (Lamarck, 1819) ♂, two of the dominant cultivated scallop species in China. Genomic *in situ* hybridization (GISH) was used to examine the chromosome constitution and variation in hybrids at early embryonic stage. The number of chromosomes in 66.38% of the metaphases was 2n = 35 and the karyotype was 2n = 3 m + 5 sm + 16 st + 11 t. After GISH, two parental genomes were clearly distinguished in hybrids, most of which comprised 19 chromosomes derived from their female parent (*Chlamys farreri*) and 16 chromosomes from their male parent (*Argopecten irradians irradians*). Some chromosome elimination and fragmentation was also observed in the hybrids.

## Introduction

Utilization of heterosis has become one of the most important strategies for increasing productivity of commercial plants and animals (Hua et al. 2003). As a major approach for this attempt, crossbreeding programs have been extensively employed in agriculture (Vyn and Tollenaar 1998, Kumar 2002, Laurila et al. 2001, Xu et al. 2003) and stockbreeding production (Kahi et al. 2000, Carrapiso et al. 2003). In aquaculture, crossbreeding has been widely developed at both research and application aspects, particularly with some teleost fish species (Rahman et al. 1995, Gross et al. 1996, Kari et al. 1997, Gharrett et al. 1999). With respect to the breeding of marine shellfish, thus far, crossbreeding methods in oyster (Stiles 1978, Menzel 1987, Scarpa and Allen 1992) and abalone (Leighton and Lewis 1982, Yan et al. 1999, Wan et al. 2001, Cai et al. 2010) have been tentatively attempted or even commercially established for aquaculture. Scallop production comprises a pillar component of Chinese shellfish aquaculture in both value and weight. Interspecific hybridization of different pairs of species have been tentatively conducted for investigating their survival potential, growth and fertility for aquaculture purpose (Bower et al. 1997, Yang et al. 2004, Bi et al. 2005, Liu and Chang 2006, Lü et al. 2006a, Lü et al. 2006b, Huang et al. 2011, Wang et al. 2011, Hu et al. 2013). However, so far, only two successful cases of scallop crossbreeding were reported. One is hybrid *Chlamys farreri* (Jones & Preston, 1904) ♀ × *Patinopecten yessoensis* (Jay, 1857) ♂, whose offspring has a high production trait as well as strong disease resistance ability (Yang et al. 2002). The other is reciprocal hybrid between *Argopecten irradians irradians* (Lamarck, 1819) and *Argopecten purpuratus* (Lamarck, 1819), and the hybrids exhibited a great increase in production traits as well as some interesting new characteristics (Wang et al. 2011).

To understand the genetic basis of heterosis, sequences of some nuclear gene and mitochondrial DNA and GISH were used to analyze the genomic constitution of scallop hybrids. Lü et al. (2006a and 2006b) reported that the chromosome number in most of the scallop hybrid between *Chlamys farreri* and *Patinopecten yessoensis* was 38, which was accordant to that of their parents. But some abnormal chromosome constitutions were found including haploid, triploid, aneuploid and some gynogenesis-like individuals. The analysis of chromosome components in scallop hybrids between *Mimachlamys nobilis* (Reeve, 1852) and *Chlamys farreri* by Huang et al. (2011) indicated that most of reciprocal hybrids contained 35 chromosomes, corresponding to the theoretical expectation of hybrids between the two species, and a few gynogenetic individuals, as well as chromosome fragmentations, aneuploids and allopolyploids were also detected in some F1 individuals. In the scallop hybrid between *Argopecten purpuratus* and *Argopecten irradians irradians* (Hu et al. 2013), GISH verified a combination of haploid genomes of duplex parents in the hybrids. The sequence of the ribosomal DNA internal transcribed spacer region (ITS) showed that the hybrid offspring not only harbored alleles from their parents but also produced some recombinant variants, which revealed some alterations in the nuclear gene of the hybrids. The mitochondrial 16S rDNA indicated a matrilineal inheritance in scallops. These progresses of genomic analysis in interspeciﬁc hybrids showed us some interesting phenomena of genomic structure in scallop hybrids.

The Zhikong Scallop, *Chlamys farreri* is a native species of Northern China. It is an important cultivated scallop species and has accounted for over 60% of the total scallop production in China. The Bay Scallop, *Argopecten irradians irradians*, was introduced from North America to Qingdao in 1982 (Zhang et al. 1986). Bay scallops grow quickly and can reach market size (50–60 mm) within a year, which is much faster than Zhikong scallops which usually take 1.5–2.0 years to reach market size. Because of the short grow-out time, bay scallops became an important marine cultured species in China. The production of bay scallops increased considerably due to severe summer mortalities of Zhikong scallops since 1997. These two species have different cytogenetic features. *Chlamys farreri* has a diploid number of 38 with a karyotype of 6m + 10cm + 22st (Wang et al. 1990), but the karyotype of *Argopecten irradians irradians* is 2n = 32 = 10st + 22t (Wang and Guo 2004). In addition, in *Chlamys farreri*, the major and minor rRNA genes had one locus each and were mapped to the same chromosome. While in *Argopecten irradians irradians*, the major rRNA genes had two loci, the minor rRNA gene had one locus, and all of these three loci were on different chromosomes (Wang and Guo 2004). With these apparent ecological and genetic differences, *Chlamys farreri* and *Argopecten irradians irradians* may be potentially useful for crossbreeding to obtain desirable scallop breeds.

We artificially carried out interspecific crossing between *Chlamys farreri* and *Argopecten irradians irradians* as an initial step of the ongoing crossbreeding project. In the present study, we reported experimental results of using GISH to verify the hybrid identity of the larvae, and documented a number of interesting patterns of karyotypic abnormalities in some hybrids.

## Material and methods

### Scallop materials

Sexually mature scallop *Chlamys farreri* ♀ and *Argopecten irradians irradians* ♂ (two years old) were obtained from Changfei Scallop Hatchery in Shandong Province, China. Artificial hybridization was carried out in the lab. The main procedures are as followed. Mature parents were induced to spawn by exposure to air for 30 min followed with a temperature shock in 20 °C seawater. Because *Argopecten irradians irradians* is hermaphroditic, sperm was filtered by a 25 µm mesh screen in order to avoid introducing eggs of *Argopecten irradians irradians*. After collection of the gametes, eggs from *Chlamys farreri* were mixed with sperms from *Argopecten irradians irradians* to produce hybrids. Hybrid larvae were reared at 20 °C and sampled at the swimming trochophore stage (approximate 20 h after fertilization) and used for chromosome preparation.

### Chromosome preparations

Following colchicine (0.01%) treatment for 2 h at room temperature, the larvae were exposed to 0.075 M KCl for about 30 min. After fixation in Carnoy’s fixative (methanol: glacial acetic acid=3:1 v/v) for 3 times (each 15 min), samples were stored at -20 °C. The fixed larvae were dissociated into fine pieces by pippetting in 50% acetic acid. The cell suspension was dropped on hot-wet glass slides and air-dried. For FISH analysis, the chromosome preparations were air-dried and preserved in a moist chamber at -20 °C until use.

### Genomic DNA extraction and labeling

Total genomic DNA was extracted from adductor muscle using traditional phenol/chloroform method described by Sambrook et al. (1989). Genomic DNA from one parent was labeled with biotin-11-dUTP by nick translation following the manufacturer’s protocol (ROCHE). The length range of probe fragments was approximately 100~600 bp. Labeled probe was purified, ethanol-precipitated and then resolved at a concentration of 5 ng/µl in a hybridization solution of 2×SSC, 50% deinoized formamide, 10% dextran sulphate and 100 µg/µl salmon testis DNA, pH 7.0. A 10-fold unlabeled blocking DNA from the other parental scallop species, which was sonicated to generate fragments of approximately 100~300 bp in length, was added into probe solution in order to block the DNA of the corresponding species.

### Genomic *in situ* hybridization

Genomic *in situ* hybridization and probe detection were performed as described by Bi and Bogart (2006) with minor modifications. Before hybridization, slides were incubated at 50 °C for about 3 h, treated with 100 µg/ml RNase A in 2×SSC at 37 °C for 30 min, washed with 2×SSC at room temperature for 15 min, and denatured in a mixture contains 75% formamide and 2×SSC for 2~3 min at 72 °C, dehydrated through a ice-cold ethanol series including 70%, 90% and 100%, 5 min each, and air-dried. Genomic DNA probe mixture was denatured for 5 min at 80 °C, followed by immediately putting on ice for at least 10 min. Probe was pre-annealed by incubating for at 32 °C 5 min prior to hybridization. The probe hybridization mix was applied to the slide and DNA-DNA in situ hybridization was carried out in a dark humid container at 37 °C for 16~18 h. Following hybridization the slides were washed twice in 2×SSC, and 50% formamide at 42 °C for 10 min, 1×SSC at 42 °C for 10 min and finally in 2×SSC at room temperature for 10 min. Biotinylated probes were detected with fluorescein isothiocyanate (FITC) conjugated avidin DCS (Cell Sorting Grade VECTOR) for 1 h at 37 °C. Chromosomes were counterstained with propidium iodide (VECTOR) for 40 min at 37 °C. Hybridization signals were detected by using Nikon epifluorescence microscope E-600 equipped with the appropriate filter sets for FITC and PI. More than 50 metaphase plates were examined by GISH.

### Image processing

Digital images were recorded using a CCD camera (COHU) and analyzed with software of Lucia - FISH Image System. The karyotype was determined from more than 10 good metaphase plates and classified according to the criteria defined by Levan et al. (1964).

## Results and discussion

**Figure 1.**
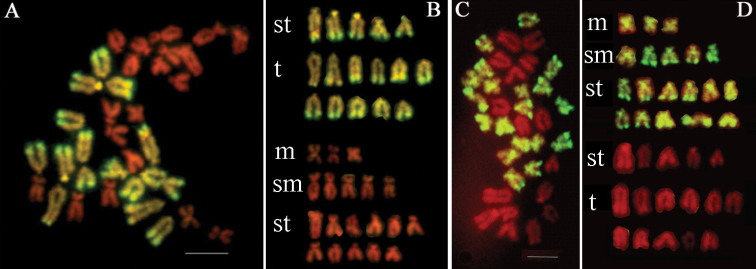
Representative metaphase chromosomes and karyotypes of F1 hybrids of *Chlamys farreri* ♀ × *Argopecten irradians irradians* ♂ examined by GISH. Chromosomes were labeled by FITC (green) and counterstained by PI (red). In (**A**, **B**), chromosomes originated from *Argopecten irradians irradians* were painted green using the labeled genomic DNA probes from *Argopecten irradians irradians*. In (**C**, **D**), chromosomes from *Chlamys farreri* were painted green using the labeled genomic DNA probes from *Chlamys farreri*. m: metacentric, sm: submetacentric, st: subtelocentric, t: telocentric. Bars = 5 µm.

The chromosome number of hybrids was determined by observing more than one hundred metaphase plates. The statistic results showed that 66.38% of 116 metaphase plates present a diploid component of 2n = 35 in the hybrids. Ten metaphase plates of hybrids were selected to measure arm length and calculate arm ratio and relative length of chromosomes. The karyotype of hybrids is 2n = 35 = 3m + 5sm + 16st + 11t. Typical mitotic spread of the hybrids was shown in Figure 1. The karyotype of *Chlamys farreri* is 2n = 38 = 6m + 10sm + 22st (Wang et al. 1990), while that of *Argopecten irradians irradians* is 2n = 32 = 10st + 22t (Wang and Guo 2004). Most of the hybrid metaphase plates had a diploid chromosome number of 35, as expected from the parental haploid complements. According to the chromosome configuration, all 3 metacentric chromosomes belonged to *Chlamys farreri*, but not *Argopecten irradians irradians*. And all 11 telocentric chromosomes, on the contrary, belonged to *Argopecten irradians irradians* but not *Chlamys farreri*. These chromosome morphological characteristics can be used for chromosome identification in the hybrid metaphases.

By using blocking DNA and pre-annealing to block homoeologous sequences, labeled genomic DNA probes from one parent could not hybridize to chromosomes from the other one. GISH effectively distinguished all chromosomes of *Chlamys farreri* and *Argopecten irradians irradians* in their hybrids, respectively. Examples of GISH results with detection of respective parental genomic DNA probes in hybrids were shown in Figure 1. FITC-labeled genomic DNA of *Argopecten irradians irradians* blocked with unlabeled *Chlamys farreri* genomic DNA was hybridized *in situ* to mitotic metaphase chromosome of the hybrids (Fig. 1A, B). At the same time, FITC-labeled genomic DNA of *Chlamys farreri* blocked with unlabeled *Argopecten irradians irradians* genomic DNA was hybridized *in situ* to mitotic metaphase chromosome of the hybrids (Fig. 1C, D). On metaphase plates, though much genome cross-hybridization existed, strong contrast could be detected between fluorescein and PI staining. The karyotype of hybrids was 2n = 3m + 5sm + 16st + 11t, which credibly proved to be a combination of haploid genomes of two parents. Of a complement of 35 chromosomes, 19 chromosomes originated from *Chlamys farreri*, whereas the remaining 16 were of *Argopecten irradians irradians* origin. Chromosome investigation is an effective method for hybrid genomic analysis. GISH is an efficient cytogenetic technique which allows chromosomes from different parents or ancestors to be distinguished. Labeled total genomic DNA from one parental species was used as a probe, and has often been found to be specific enough to mark the chromosomes from the other parent. Using this technique in hybrids, it is possible to determine the genome origin of paired and unpaired chromosomes in metaphase. GISH has been successfully used in analysis of genome origin and organization of the hybrid plant (Brysting et al. 2000, Gavrilenko et al. 2001, Falistocco et al. 2002), fish (Fujiwara et al. 1997; Sakai et al. 2007; Ráb et al. 2008) and shellfish (Cai et al. 2010; Lü et al. 2006b; Huang et al. 2011; Hu et al. 2013).

In most metaphases, hybridization signals were not uniform along chromosomes. Some strong signals are located on telomeric region of long arms and centromeric regions in *Chlamys farreri* (Fig. 1A), and on the telomeric region of almost all long arms and two short arms in *Argopecten irradians irradians* (Fig. 1C). These uneven signals along chromosomes indicated that some repetitive sequences were located on these regions, which was revealed by FISH using species-specific satellite probes in *Chlamys farreri* (Zhang et al. 2008, Hu et al. 2011, Huang et al. 2011). In *Argopecten irradians irradians*, these strong signal locations were accordance with the heterochromatic regions on chromosomes revealed by C-bands, DAPI-bands and FISH using *C_0_t*-1 DNA probes (Huang et al. 2007, Hu et al. 2011). The heterochromtic regions were found mainly in telomeric and centromeric regions by some banding methods in mollusk including scallops (Insua et al. 1998, López-Piñón et al. 2005, Huang et al. 2007), mussels (Martínez-Expósito et al. 1997, Torreiro et al. 1999, Pérez-García et al. 2011) and oysters (Li and Havenhand 1997, Wang et al. 2001, Cross et al. 2005). In addition, the nonuniform distribution of the signals reflected genomic repetitive DNAs to the chromosomes by self-GISH, which were observed in fishes (Targino et al. 2009), plants (She et al. 2007), insects (Pita et al. 2014) and mammals (Suarez-Villota et al. 2012). In *Argopecten irradians irradians*, the short arm of two subtelocentric chromosomes showed strong signals after GISH (Fig. 1A). The morphology of these two chromosomes was similar with those two pairs of chromosomes with NORs verified by silver staining and FISH using 18S-28S rDNA probes (Huang et al 2007). The strong signals in NORs were also found in heterochromatic region by self-GISH in plants (She et al. 2007). So we speculated the strong signal regions on short arm of two chromosomes in *Argopecten irradians irradians* were the nucleolus organizer regions (NORs).

**Figure 2.**
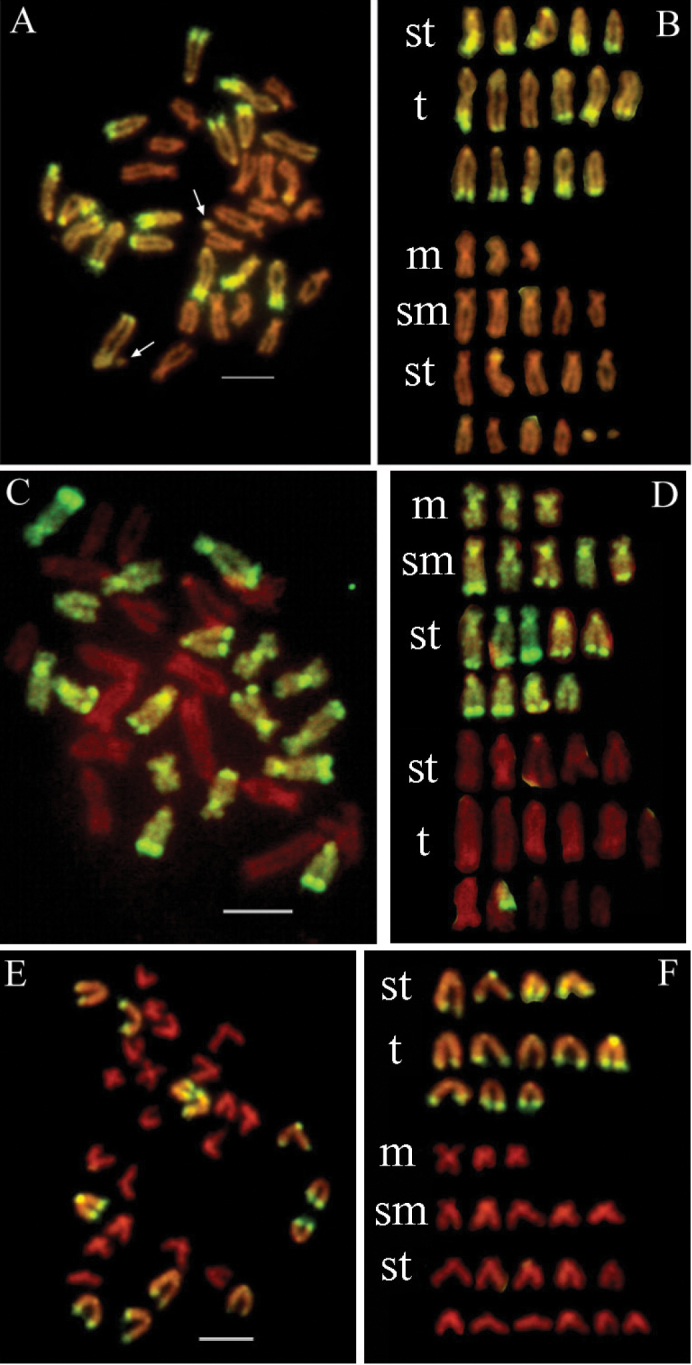
Examples of chromosome fragments (**A, B**) and chromosome eliminations (**C, D, E, F**) in the F1 hybrids. In (**A, B**), chromosome fragments originated from *Chlamys farreri* were marked with arrows. In (**C, D**), some chromosomes from *Chlamys farreri* eliminated in the metaphase spread. In (**E, F**), some chromosomes from *Argopecten irradians irradians* eliminated in the metaphase spread. In (**A, B, E, F**), chromosomes were labeled by GISH using *Argopecten irradians irradians* genomic DNA probes (green). In (**C, D**), chromosomes were labeled by GISH using *Chlamys farreri* genomic DNA probes (green). Bars = 5 µm.

During the examination, we also found some metaphases containing chromosome fragments and chromosome elimination. Chromosome fragments were found to originate from *Chlamys farreri* (Fig. 2A, B) in only two metaphases. This phenomenon of chromosome fragments was not reported in other scallop interspecific hybridization. In addition, we found chromosomes derived from *Chlamys farreri* were eliminated in 17.24% metaphases (Fig. 2C, D), which was apparently higher than those from *Argopecten irradians irradians* in 9.32% metaphases (Fig. 2E, F). In Table 1, totally 33.62% metaphases were aneuploid, much higher than the intraspecific cross groups 15.6% for *Chlamys farreri*, indicating the instability of the hybrid genome (Huang et al. 2011). Chromosome abnormality is known to be one of the causes for hybrid inviability in some salmonid interspecific hybrids, which is induced by a possible incompatibility between paternal genome and maternal cytoplasm (Fujiwara et al. 1997). Chromosome elimination is observed in natural hybrids of insects such as *Nasonia* (Ashmead, 1904) (Breeuwer and Werren 1990, Reed and Werren 1995). We speculated that the observed chromosome elimination in scallop hybrids was influenced by the ratio or property of parental nuclear genomes and cytoplasms, where chromosomes from one parent were always eliminated by their asynchronous behaviors during mitosis.

**Table 1.** Chromosome number of hybrids (*Chlamys farreri* ♀ × *Argopecten irradians irradians* ♂).

Chromosome number										
	≤30	31	32	33	34	35	36	37	≥38	Total
Number of analyzed metaphases	2	3	9	10	7	77	7	1	0	116
Frequency (%)	1.72	2.59	7.76	8.62	6.03	66.38	6.04	0.86	0	100
